# Evaluation of effectiveness of concentrated growth factor on osseointegration

**DOI:** 10.1186/s40729-017-0069-3

**Published:** 2017-03-03

**Authors:** Cagasan Pirpir, Onur Yilmaz, Celal Candirli, Emre Balaban

**Affiliations:** Faculty of Dentistry, Department of Oral and Maxillofacial Surgery, Karadeniz Technical University, Trabzon, Turkey

**Keywords:** Dental implants, Growth factors, Osseointegration, Osteogenesis, Wound healing

## Abstract

**Background:**

Growth factor-containing products have been reported to increase implant stability and accelerate osseointegration. Concentrated growth factor (CGF) can be used for this purpose with the growth factors it contains. The aim of this study is to assess the effect of CGF on implant stability and osseointegration.

**Methods:**

Twelve patients with maxillary anterior toothless were included in the study. Implant cavities prepared in the study group were covered with CGF membrane before implant placement, but conventional implant placement was performed in the control group. Resonance frequency measurements were performed with the Osstell device intra-operatively, post-operatively, at the 1st week, and at the 4th week.

**Results:**

The mean ISQ values were found to be 79.40 ± 2.604 for the study group and 73.50 ± 5.226 for the control group at 1st week, 78.60 ± 3.136 for the study group and 73.45 ± 5.680 for the control group at 4th week. The differences between the groups were statistically significant (*p* < 0.05).

**Conclusions:**

It was observed that the concentrated growth factor had positive effects on implant stabilization. The ISQ measurements at week 1 and week 4 were notably higher in the study group. Application of this material seems to accelerate osseointegration.

## Background

Osseointegration of dental implants is important for long-term success and stability. There is no standardization in terms of the time of osseointegration and the timing of prosthetic loading. This process varies between 0–6 months [1]. Various strategies are being explored to shorten this period. Changes in implant surface properties and design have increased primer stability and helped the peri-implant tissue remain healthy. These changes have aimed to increase bone-implant surface connectivity and accelerate healing. Another method of accelerating osseointegration is the modulation of healing after the placement of the implant [2]. This modulation, in turn, can be achieved by bioactive molecules that increase osteoblastic differentiation and accelerate bone healing around the implant [2].

Growth factors are bioactive proteins that control the wound healing process. The platelet-containing preparations derived from human blood contain many growth factors such as bone morphogenetic protein (BMP), platelet-derived growth factor (PDGF), insulin-like growth factor (IGF), vascular endothelial growth factor (VEGF), transforming growth factor-β1 (TGF-β1), and transforming growth factor-β2 (TGF-β2), which also play a key role in bone healing [3–5]. These growth factors attract the undifferentiated mesenchymal cells to the wound site, thus facilitating angiogenesis, chemotaxis, and cell proliferation [2].

Various platelet concentrates such as platelet-rich plasma (PRP), platelet-rich fibrin (PRF), and concentrated growth factor (CGF) are used to reconstruct bone defects [6]. PRF has been shown to have very successful results in tissue engineering in many studies [7–9]. Furthermore, a study by Sohn et al. has shown the higher regeneration capacity and multipurpose use of CGF in 2009 [10].

This preparation’s potential is because it contains growth factor-containing fibrin network; it contains fibroblast, platelet, leukocyte, and endothelial cell for angiogenesis and tissue remodeling; and it provides matrix for cell migration [11]. Platelets, in particular, contain biologically active proteins at high concentrations and support healing, growth, and cell morphogenesis [12–14].

That the implant has sufficient stability after placement is important for providing the necessary bone formation around the implant and for the optimal distribution of functional forces at the implant-bone interface during healing [15–17].

It can be said that resonance frequency analysis (RFA) is a very important tool for tracking the osseointegration process [18, 19]. RFA is a technique that allows tracking the changes in stability not only during implant placement but also during healing and later periods [20].

Growth factor-containing products have been shown to accelerate bone healing and osseointegration [2, 4]. In this study, it is aimed to evaluate the effect of CGF on implant stability. Based on the results obtained in the study, it will be possible to reduce the amount of time required for osseointegration.

## Methods

This study was conducted in compliance with the principles of the Declaration of Helsinki, and approval of the ethics committee required for the study was obtained from the Ethics Committee of the Karadeniz Technical University (2015/21). The procedures to be performed were explained in detail and patients signed the consent forms. The study was carried out on individuals who applied to Karadeniz Technical University, Faculty of Dentistry, Department of Oral and Maxillofacial Surgery to get dental implants for upper jaw tooth deficiencies. In terms of standardization, only patients with implants applied to maxillary anterior and premolar region were included in the study. Cylindrical implants were used in each patient. The diameter of the implant was 3.5 or 4.0 mm, and the length was 10 mm. In patients who underwent tooth extraction, implants were placed 6 months after extraction. Patients rehabilitated with a fixed prosthesis, such as a single crown or bridge, were included in the study. Patients included in the study were randomly assigned to two groups: study and control groups.

Exclusion criteria were identified as:

- Presence of systemic diseases preventing implantation
- Having blood disease to prevent centrifugation
- Previous implantation or augmentation of the same region
- The need for additional bone augmentation procedures (such as maxillary sinus augmentation, distraction osteogenesis)
- Allergy to one of the materials to be used during operation
- Pregnancy
- Smoking

The implanted regions were evaluated preoperatively with panoramic radiography and computed tomography (CT) images. In the study group, the socket walls were laid with CGF membrane while the implant surfaces were washed with the thrombocyte-deprived part of the tube. No different procedure was done to the implants and socket in the control group.

### CGF preparation

**Fig. 1:**
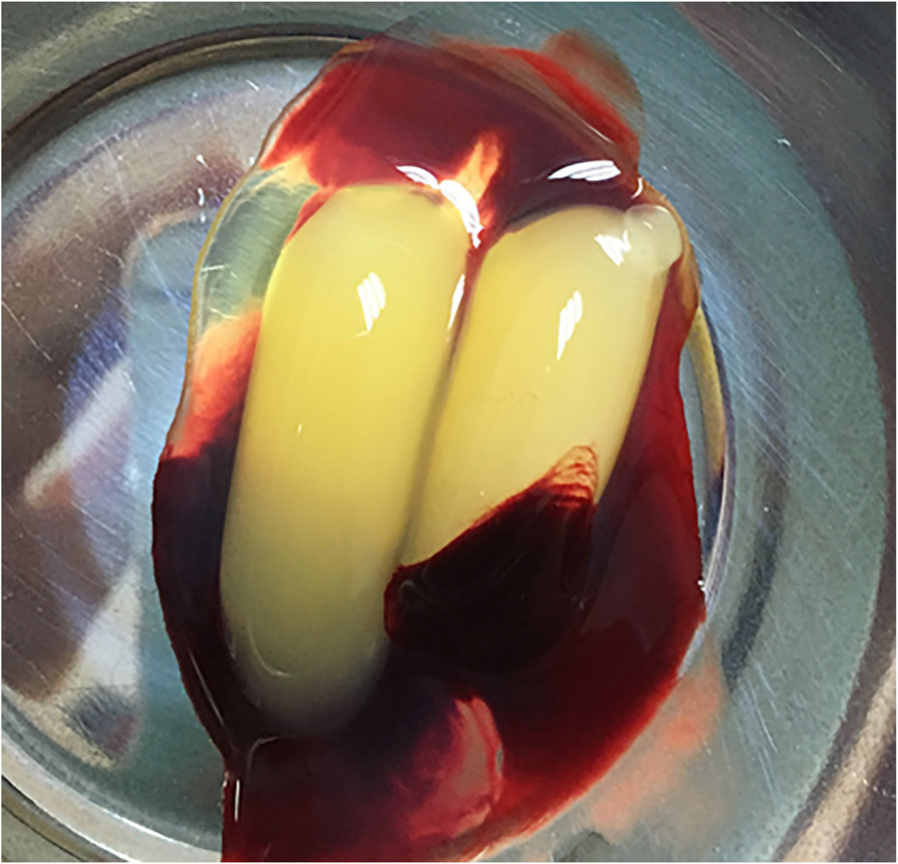
CGF was obtained after centrifugation

A standard, disposable, 10-ml non-anticoagulant tube and a matching centrifuge device (MEDIFUGE, Silfradentsrl, S. Sofia, Italy) were used. Intravenous blood samples from the patients were placed in centrifuge tubes without anticoagulants and accelerated for 30 s, centrifuged at 2700 rpm for 4 min, 2400 rpm for 4 min, 2700 rpm for 4 min, and 3000 rpm for 3 min, and decelerated for 36 s to stop. All of these acceleration and deceleration processes are adjusted automatically due to the centrifugal device’s feature. Three layers were observed in the tube: red blood cell layer at the bottom, platelet-deprived plasma layer (without cell) at the top, and fibrin gel with concentrated growth factor and platelet aggregation in the middle. First, the uppermost platelet-deprived fraction was removed with a sterile syringe. The layer in the form of a membrane containing the concentrated growth membrane was held with the aid of a hemostatic clamp, separated from the red blood cell layer by cutting with a pair of scissors and then pressed to form a membrane (Fig. 1).

### Surgical procedure

**Fig. 2:**
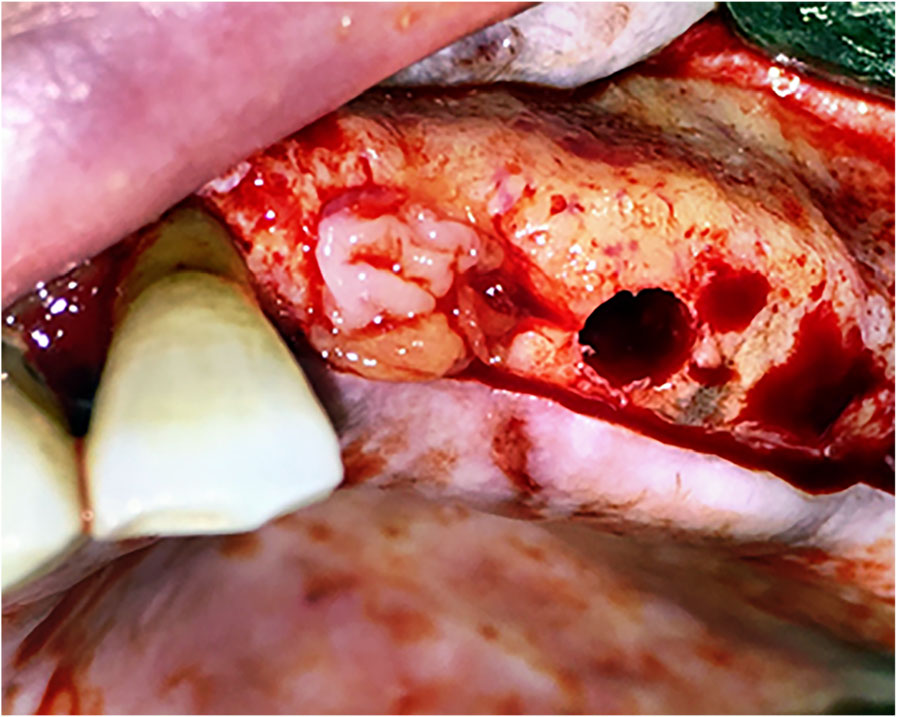
CGF membrane was applied in study group implant sockets

**Fig. 3:**
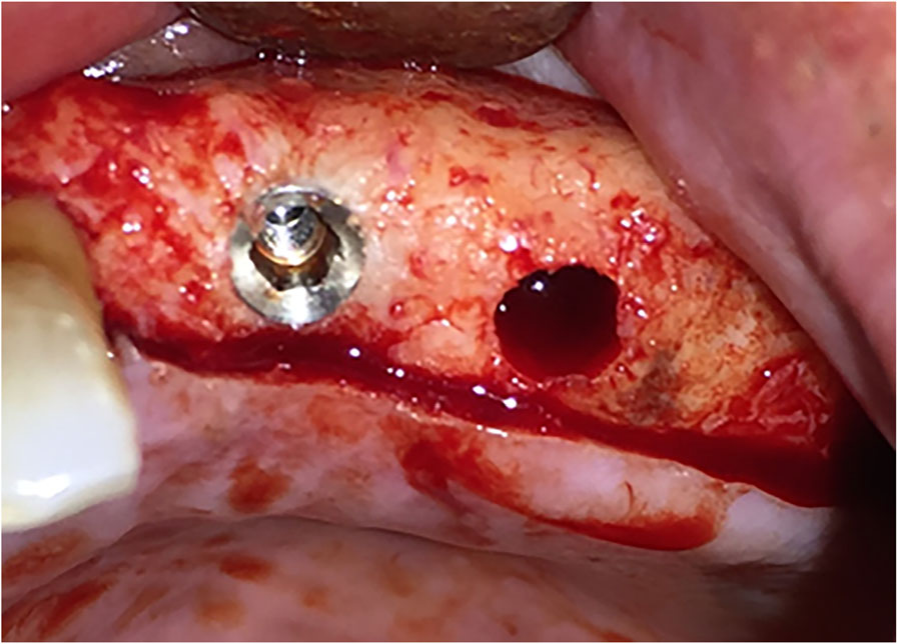
Implants were placed after application of CGF membrane

All surgical procedures were performed under local anesthesia by the same surgeon. A full-thickness mucoperiosteal flap was removed by incision on the alveolar crest. Implant cavities were prepared according to the surgical protocol of the Bego Semados implant system (BEGO Implant Systems GmbH & Co. KG, Bremen, Germany). The final osteotomy diameters were the same as the placed implants. In the study group, the implant cavity walls were laid with CGF membranes all around (Fig. 2). The implants in the study group were irrigated with CGF fluid on their surface and placed in the corresponding socket (Fig. 3). The implants representing the control group were placed in the corresponding socket without any additional procedure. The implant surgery was completed in one session by attaching gingival formers to the implants in both groups. Gingival formers were not covered with soft tissue and mucoperiosteal flaps were sutured with 3/0 silk suture material.

The patients were recommended to apply cold compresses after the surgery. Patients were prescribed antibiotic (amoxicillin + clavulanic acid combination 1000 mg 2 × 1), analgesic (arveles tablet, 25 mg 2 × 1), and antiseptic mouthwash (0.2% chlorhexidine gluconate mouthwash, 3 × 1) for 1 week. The patients were controlled at the 1st week, 4th week and 12th week.

### Resonance frequency analysis measurements

The stability of implants by RFA was done using Osstell instrument (Integration Diagnostics, Goteborg, Sweden) with Smartpeg™ (Integration Diagnostics, Goteborg, Sweden), a transducer suitable for implants. The measurement result is obtained from the resonance frequency values by an arithmetic algorithm and given as the ISQ (implant stability quotient) value. At measurement time, the transducer, Smartpeg™, was placed in the region where the prosthetic part is located in the implants.

For measurements, the Osstell™ probe was approximated to the Smartpeg™ from the buccal, palatal, mesial, and distal sides. For each implant, resonance frequency values were obtained in four different directions. The ISQ values were averaged to determine the mean ISQ value for each implant. The ISQ values of the implants were measured during the operation, at the first week and at the fourth week after the operation.

### Statistical analysis

Independent sample *t* test was applied between the two groups by taking the differences between the data obtained in these periods. Two-way ANOVA and Fisher’s LSD test was used for evaluating the associations among group and insertion torque.

A value of *p* < 0.05 was considered statistically significant. All evaluations were performed with Windows SPSS (version 17.0, IBM Corp., NY, USA) software.

## Results

The study includes 12 patients (5 males, 7 females). Patients participating in the study are between 20–68 years of age and the mean age is 44 years. A total of 40 implants were placed, 20 of these were included in the study group (50%), and the other 20 were included in the control group (50%). Twenty-one implants were placed in type 2 bone, 19 implants in type 3 bone (Table 1). The distribution of gender, installed implant diameter, and bone quality between the control group and the experimental group did not show any statistically significant difference. No complications occurred during the postoperative period.

**Table 1:** Demographic data of patients

Case no.	Age	Sex	Group	Implant number
1	20	F	Study	1
2	28	M	Control	3
3	35	F	Study	4
4	32	F	Study	4
5	60	M	Control	5
6	64	F	Study	5
7	52	F	Study	5
8	34	M	Study	1
9	45	F	Control	3
10	48	F	Control	2
11	42	M	Control	3
12	68	F	Control	4

At the implant placement, the average torque for study group was 31,700 ± 2,696 Ncm and for control group was 30,55 ± 2,163 Ncm; there was no significant difference between the two groups (*p* = 0.098).

The mean ISQ values measured after the placement of the implants were 75.75 ± 5.552 for the control group and 78.00 ± 2.828 for the study group. There was no statistically significant difference between control and study groups in terms of initial ISQ values (*p* > 0.05).

**Fig. 4:**
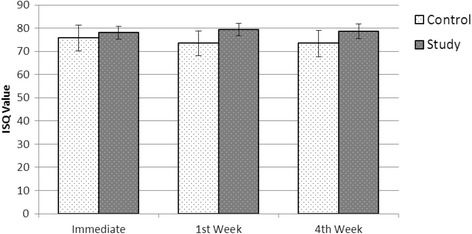
Comparative illustration of mean ISQ values

The postoperative ISQ values were found to be 79.40 ± 2.604 for the study group and 73.50 ± 5.226 for the control group at 1st week, 78.60 ± 3.136 for the study group and 73.45 ± 5.680 for the control group at 4th week (Table 2). It was determined that the differences between the groups were statistically significant (*p* < 0.05) and the ISQ measurements at week 1 and week 4 were notably higher in the study group (Fig. 4).

**Table 2:** Mean ISQ values in the study and control groups

	Control group	Study group
Immediate	75.75 ± 5.552	78.00 ± 2.828
1st week	73.50 ± 5.226	79.40 ± 2.604
4th week	73.45 ± 5.680	78.60 ± 3.136

The increase and decrease rates of ISQ values between periods were evaluated by paired *t* test. Immediate postoperative measurements and 1st week measurements were compared. An increase of 1.40 ± 1.847 was observed in the study group while there was a decrease of 2.25 ± 1.713 in the control group; this difference was statistically significant (*p* < 0.001). When the difference between the immediate postoperative measures and the 4th week measurements were evaluated, an increase of 0.60 ± 2.798 was observed in the study group while a decrease of 2.30 ± 2.774 was observed in the control group; this difference was statistically significant (*p* = 0.002). When the difference between the measurements of the control group and the study group at 1st week and 4th week was evaluated, a decrease of 0.05 ± 1.572 was observed in the control group while a decrease of 0.80 ± 2.215 was observed in the study group; this difference was not statistically significant (*p* = 0.224) (Table 3).

**Table 3:** Mean ISQ value changes between study and control groups

	Control group	Study group
Immediate–1st week	−2.25 ± 1.713	1.40 ± 1.847
Immediate–4th week	−2.30 ± 2.774	0.60 ± 2.798
1st Week–4th week	−0.05 ± 1.572	−0.80 ± 2.215

## Discussion

Implant stability is one of the important parameters that assess the loading time and dental implant success. Investigators have recommended that implants with ISQ < 49 measured when placed should not be loaded during the 3-month healing period; implants with ISQ ≥ 54 may be loaded. It is emphasized that implants with low primer stabilization value should be waited to reach a stabilization value adequate for prosthetic loading and that they must be protected against mechanical trauma and infection during this time [21].

In some studies, there is a meaningful reduction in ISQ values measured sometime after the placement of implants [22–24]. Huwiler et al. [24] have indicated that this reduction occurs during 2nd–4th week period while Monov et al. [23] have stated that it occurs as early as 4 days after the operation. In this study also, a decrease in the 1st week ISQ values was observed in the control group. Investigators have suggested that this decrease in stability values and subsequent increase are due to remodeling occurring during bone healing [22–24]. In the implants in the study group, an increase or stability was observed. A statistically significant difference was found between the study and control groups in each period of analysis. This suggests that CGF administration affects the implant primer stability by accelerating the osseointegration process.

Growth factors indicate that they accelerate tissue healing when they function effectively. Studies in the literature have reported that thrombocytes secrete growth factors from α-granules and that these releasing growth factors promote collagen synthesis. Increased collagen synthesis is thought to play a role in increasing soft tissue resistance and in the initiation of callus formation in bone tissue [14, 25–27].

Thrombocytes (platelets) also coexist with other thrombocytes, allowing the fibrin network to remain stable [28]. Within this stable, fibrin clot formation are chemical attractants in surrounding cells such as cell adhesion proteins, thrombocytes, and plasma growth factor; some of these mitogens are related to direct osteogenic cell function [29].

Introduced in 1998 by Marx, PRP is used in oral and maxillofacial surgeries to speed up the recovery of grafts in bone-grafted areas [14, 26–30]. Although many studies have shown that platelet-rich plasma affects bone healing positively, the results of some other studies suggest otherwise [31, 32].

In recent years, the platelet-rich fibrin (PRF) was described by Choukroun as a second-generation platelet concentrate [33]. PRF is defined as leukocyte and platelet-rich fibrin biomaterial. PRF is used to accelerate healing in maxillary sinus augmentation, socket healing after tooth extraction, filling of the cyst cavity, treatment of furcation defects in periodontology, and soft tissue injuries [34].

The positive effects of blood products on healing have also triggered the development of products in different concentrations. One of these products, the concentrated growth factor (CGF), was defined by Sacco in 2006 [35]. CGF also has its own centrifugal technique in a manner similar to PRF. A longer and denser fibrin matrix with higher growth factor content was obtained by the different centrifugation technique [35].

Regional CGF administration increases FGF-β or VEGF release, which plays an active role in angiogenesis, as well as enhancing neutrophil migration by performing integrin release. It has also been shown that CGF contains such growth factors and CD34-positive cells [35]. It has been reported that CD34-positive cells in the cells also provide angiogenesis, neovascularization, and vascular continuity [36, 37].

In an animal study, CGF, PRF, and PRP were placed separately in the defects formed in the rabbit skull in the study group; the defects were left empty in the control group. Histomorphometric analysis revealed statistically significant differences between control and study groups in the growth of new bone formation at 6 and 12 weeks. In the study group, the greatest bone formation was observed in the CGF-treated group but this difference was not statistically significant [38]. In a study by Takeda et al. performed on rats, it was observed that cell proliferation and osteoblastic differentiation in the cell culture from the CGF-treated group was significantly higher than in the other groups [39].

In a study by Monov et al. using PRP around the implant, a higher stability value was obtained in the study group during the early recovery period (6 weeks) although difference between the groups was not statistically significant [23]. Kim et al. reported in a study that there was a statistically significant increase in bone-implant contact with PRP administration in the vicinity of the implant [40].

Based on all these results, it can be said that CGF and PRF accelerated the implant osseointegration process and affected the stabilization values positively. It has been reported that CGF contains more growth factors than other platelet preparations [35]. It was observed in our study that CGF has favorable effects on implant healing period. There is no publication on the effect of CGF on the stability of dental implants during healing period. For this reason, we investigated the effect of CGF on the stability of dental implants in our study.

## Conclusions

Considering this data, it appears that application of CGF enhanced stability of implants and accelerated osseointegration in the early period. CGF has positive effects on the ISQ value at the first week and fourth week. Further laboratory studies are needed to demonstrate the positive effects of blood products on the osseointegration process at the histopathological level.

## Abbreviations

BMP: Bone morphogenetic protein
CGF: Concentrated growth factor
CT: Computed tomography
IGF: Insulin-like growth factor
PDGF: Platelet-derived growth factor
PRF: Platelet-rich fibrin
PRP: Platelet-rich plasma
RFA: Resonance frequency analysis
TGF-β1: Transforming growth factor-β1
TGF-β2: Transforming growth factor-β2
VEGF: Vascular endothelial growth factor

## References

[1] Raghavendra S, Wood MC, Taylor TD (2005). Early wound healing around endosseous implants: a review of the literature. Int J Oral Maxillofac Implants.

[2] Oncu E, Bayram B, Kantarci A, Gulsever S, Alaaddinoglu EE (2016). Positive effect of platelet rich fibrin on osseointegration. Med Oral Patol Oral Cir Bucal.

[3] Anitua E (1999). Plasma rich in growth factors: preliminary results of use in the preparation of future sites for implants. Int J Oral Maxillofac Implants.

[4] Anitua E, Andia I, Ardanza B, Nurden P, Nurden AT (2004). Autologous platelets as a source of proteins for healing and tissue regeneration. Thromb Haemost.

[5] Anitua E, Orive G, Pla R, Roman P, Serrano V, Andia I (2009). The effects of PRGF on bone regeneration and on titanium implant osseointegration in goats: a histologic and histomorphometric study. J Biomed Mater Res A.

[6] Bhanot S, Alex JC (2002). Current applications of platelet gels in facial plastic surgery. Facial Plast Surg.

[7] Coetzee JC, Pomeroy GC, Watts JD, Barrow C (2005). The use of autologous concentrated growth factors to promote syndesmosis fusion in the agility total ankle replacement. A preliminary study. Foot Ankle Int.

[8] Rutkowski JL, Thomas JM, Bering CL, Speicher JL, Radio NM, Smith DM (2008). Analysis of a rapid, simple, and inexpensive technique used to obtain platelet-rich plasma for use in clinical practice. J Oral Implantol.

[9] Dohan Ehrenfest DM, de Peppo GM, Doglioli P, Sammartino G (2009). Slow release of growth factors and thrombospondin-1 in Choukroun’s platelet-rich fibrin (PRF): a gold standard to achieve for all surgical platelet concentrates technologies. Growth Factors.

[10] Sohn DS, Heo JU, Kwak DH, Kim DE, Kim JM, Moon JW (2011). Bone regeneration in the maxillary sinus using an autologous fibrin-rich block with concentrated growth factors alone. Implant Dent.

[11] Gassling VL, Acil Y, Springer IN, Hubert N, Wiltfang J (2009). Platelet-rich plasma and platelet-rich fibrin in human cell culture. Oral Surg Oral Med Oral Pathol Oral Radiol Endod.

[12] Nurden AT, Nurden P, Sanchez M, Andia I, Anitua E (2008). Platelets and wound healing. Front Biosci.

[13] Anitua E, Sanchez M, Zalduendo MM, de la Fuente M, Prado R, Orive G (2009). Fibroblastic response to treatment with different preparations rich in growth factors. Cell Prolif.

[14] He L, Lin Y, Hu X, Zhang Y, Wu H (2009). A comparative study of platelet-rich fibrin (PRF) and platelet-rich plasma (PRP) on the effect of proliferation and differentiation of rat osteoblasts in vitro. Oral Surg Oral Med Oral Pathol Oral Radiol Endod.

[15] Meredith N (1998). A review of nondestructive test methods and their application to measure the stability and osseointegration of bone anchored endosseous implants. Crit Rev Biomed Eng.

[16] Salvi GE, Lang NP (2004). Diagnostic parameters for monitoring peri-implant conditions. Int J Oral Maxillofac Implants.

[17] Atsumi M, Park SH, Wang HL (2007). Methods used to assess implant stability: current status. Int J Oral Maxillofac Implants.

[18] Meredith N (1998). Assessment of implant stability as a prognostic determinant. Int J Prosthodont.

[19] Sennerby L, Meredith N (1998). Resonance frequency analysis: measuring implant stability and osseointegration. Compend Contin Educ Dent.

[20] Quesada-Garcia MP, Prados-Sanchez E, Olmedo-Gaya MV, Munoz-Soto E, Gonzalez-Rodriguez MP, Valllecillo-Capilla M (2009). Measurement of dental implant stability by resonance frequency analysis: a review of the literature. Med Oral Patol Oral Cir Bucal.

[21] Nedir R, Bischof M, Szmukler-Moncler S, Bernard JP, Samson J (2004). Predicting osseointegration by means of implant primary stability. Clin Oral Implants Res.

[22] Barewal RM, Oates TW, Meredith N, Cochran DL (2003). Resonance frequency measurement of implant stability in vivo on implants with a sandblasted and acid-etched surface. Int J Oral Maxillofac Implants.

[23] Monov G, Fuerst G, Tepper G, Watzak G, Zechner W, Watzek G (2005). The effect of platelet-rich plasma upon implant stability measured by resonance frequency analysis in the lower anterior mandibles. Clin Oral Implants Res.

[24] Huwiler MA, Pjetursson BE, Bosshardt DD, Salvi GE, Lang NP (2007). Resonance frequency analysis in relation to jawbone characteristics and during early healing of implant installation. Clin Oral Implants Res.

[25] Kroese-Deutman HC, Vehof JW, Spauwen PH, Stoelinga PJ, Jansen JA (2008). Orthotopic bone formation in titanium fiber mesh loaded with platelet-rich plasma and placed in segmental defects. Int J Oral Maxillofac Surg.

[26] Hsu CW, Yuan K, Tseng CC (2009). The negative effect of platelet-rich plasma on the growth of human cells is associated with secreted thrombospondin-1. Oral Surg Oral Med Oral Pathol Oral Radiol Endod.

[27] Shen YX, Fan ZH, Zhao JG, Zhang P (2009). The application of platelet-rich plasma may be a novel treatment for central nervous system diseases. Med Hypotheses.

[28] Lam WA, Chaudhuri O, Crow A, Webster KD, Li TD, Kita A (2011). Mechanics and contraction dynamics of single platelets and implications for clot stiffening. Nat Mater.

[29] Gruber R, Karreth F, Kandler B, Fuerst G, Rot A, Fischer MB (2004). Platelet-released supernatants increase migration and proliferation, and decrease osteogenic differentiation of bone marrow-derived mesenchymal progenitor cells under in vitro conditions. Platelets.

[30] Choi BH, Zhu SJ, Kim BY, Huh JY, Lee SH, Jung JH (2005). Effect of platelet-rich plasma (PRP) concentration on the viability and proliferation of alveolar bone cells: an in vitro study. Int J Oral Maxillofac Surg.

[31] Mooren RE, Hendriks EJ, van den Beucken JJ, Merkx MA, Meijer GJ, Jansen JA (2010). The effect of platelet-rich plasma in vitro on primary cells: rat osteoblast-like cells and human endothelial cells. Tissue Eng Part A.

[32] Aghaloo TL, Moy PK, Freymiller EG (2002). Investigation of platelet-rich plasma in rabbit cranial defects: a pilot study. J Oral Maxillofac Surg.

[33] Choukroun J, Diss A, Simonpieri A, Girard MO, Schoeffler C, Dohan SL (2006). Platelet-rich fibrin (PRF): a second-generation platelet concentrate. Part IV: clinical effects on tissue healing. Oral Surg Oral Med Oral Pathol Oral Radiol Endod.

[34] Prakash S, Thakur A (2011). Platelet concentrates: past, present and future. J Maxillofac Oral Surg.

[35] Rodella LF, Favero G, Boninsegna R, Buffoli B, Labanca M, Scari G (2011). Growth factors, CD34 positive cells, and fibrin network analysis in concentrated growth factors fraction. Microsc Res Tech.

[36] Ademokun JA, Chapman C, Dunn J, Lander D, Mair K, Proctor SJ (1997). Umbilical cord blood collection and separation for haematopoietic progenitor cell banking. Bone Marrow Transplant.

[37] Majka M, Janowska-Wieczorek A, Ratajczak J, Ehrenman K, Pietrzkowski Z, Kowalska MA (2001). Numerous growth factors, cytokines, and chemokines are secreted by human CD34(+) cells, myeloblasts, erythroblasts, and megakaryoblasts and regulate normal hematopoiesis in an autocrine/paracrine manner. Blood.

[38] Kim TH, Kim SH, Sandor GK, Kim YD (2014). Comparison of platelet-rich plasma (PRP), platelet-rich fibrin (PRF), and concentrated growth factor (CGF) in rabbit-skull defect healing. Arch Oral Biol.

[39] Takeda Y, Katsutoshi K, Matsuzaka K, Inoue T (2015). The effect of concentrated growth factor on Rat bone marrow cells in vitro and on calvarial bone healing in vivo. Int J Oral Maxillofac Implants.

[40] Kim SG, Chung CH, Kim YK, Park JC, Lim SC (2002). Use of particulate dentin-plaster of Paris combination with/without platelet-rich plasma in the treatment of bone defects around implants. Int J Oral Maxillofac Implants.

